# In Vitro Antioxidant and Bactericidal Efficacy of 15 Common Spices: Novel Therapeutics for Urinary Tract Infections?

**DOI:** 10.3390/medicina55060289

**Published:** 2019-06-19

**Authors:** Suresh Mickymaray, Mohammed Saleh Al Aboody

**Affiliations:** Department of Biology, College of Science, Al-Zulfi, Majmaah University, Majmaah 11952, Riyadh region, Saudi Arabia; m.alaboudi@mu.edu.sa

**Keywords:** spices, uropathogens, MIC, MBC, antioxidant, antibacterial activity

## Abstract

*Background and Objectives*: Bacterial urinary tract infection (UTI) is the most common ailment affecting all age groups in males and females. The commercial antibiotics usage augments antibiotics resistance and creates higher recurrence rates of such communal infections. Hence, this study is aimed at investigating the antibacterial and antioxidant potentials of 15 common spices against 11 UTI-causing bacterial pathogens. *Materials and Methods*: The antioxidant potential of the methanolic extracts was analyzed as contents of total phenols and flavonoids; radical scavenging, total reducing power, the ferric reducing power assay. Urinary pathogens were subjected to spice extracts to investigate antibacterial assays. *Results*: Preliminary phytochemical study of spices was performed to find those containing alkaloids, flavonoids, phenolic compounds, and steroids that can be recognized for their noteworthy bactericidal effects. The outcome of the antioxidative potential from the four methods demonstrated the sequence of potent antioxidant activity: *Acorus calamus >Alpinia galanga* > *Armoracia rusticana > Capparis spinosa* > *Aframomum melegueta.* The total polyphenols and flavonoids in the studied species positively correlated with their antioxidant properties. The four most effective spices (*A. calamus*, *A. galanga*, *A. rusticana*, and *C. spinosa*) had a zone of inhibition of at least 22 mm. *A. calamus*, *A. melegueta*, and *C. spinosa* had the lowest minimum inhibitory concentration (MIC) value against *Enterobacter aerogenes*, *Staphylococcus aureus* and *Proteus mirabilis*. All 15 spices had the lowest minimum bactericidal concentration (MBC) value against most of the pathogenic bacteria. *Conclusion*: The four highly potent and unique spices noted for the in vitro control of UTI-causing pathogens could be pursued further in the development of complementary and alternative medicine against UTI-causing pathogens.

## 1. Introduction

Urinary tract infection (UTI) is the most common among bacterial infections and responsible for morbidity and very serious illnesses in individuals of all age groups of both sexes [1]. Urinary tract infection normally occurs four times more often in men than in women between the ages of 16 and 35 years [2]. About 10% of females usually get UTI yearly once, and more than 40 to 60% have an infection at some point in their lives [3]. Nearly 80% of all UTIs are largely caused by sexual contact in premenopausal female; however, the other etiologies of UTIs are urinary incontinence, menopause, and conception. While UTIs may be instigated by fungi, viruses, and parasites that colonize the urinary tract, the predominant causative agents are bacteria [4]. These UTI-infecting bacteria move into the urinary bladder and urethra instigating inflammation, intolerable discomfort, and hazy urine associated with nocturia and hematuria that hinder urine output [5]. The most common causative agents for UTIs are uropathogenic organisms, including *Escherichia coli*, *Proteus vulgaris, Enterococcus faecalis*, *Staphylococcus saprophyticus, Staphylococcus aureus, Acinetobacter baumannii*, *Citrobacter freundii*, *Klebsiella pneumoniae*, *Proteus mirabilis*, *Enterobacter aerogenes*, *Pseudomonas aeruginosa*, and *Candida* species [4]. Medications such as pivemecillinam, trimethoprim-sulphamethoxazole, nitrofurantoin, fosfomycin trometamol, fluoroquinolone, and ciprofloxacin are among the commonly prescribed forms of suggested management of UTIs. Nevertheless, the augmentation of antibiotic resistance and higher recurrence rates of such common infections have a great impact on our society [6,7]. For centuries, plants have been used as traditional and alternative medicine around the world as drugs and therapies for several ailments and infectious diseases [8,9]. Numerous studies have shown that the synergistic effect of phytochemicals plays a significant role in the use of plant extracts as antibacterial agents [10,11]. 

Spices have been consumed as food and medicine and used as flavoring materials as well as food preservatives since time immemorial [12]. They are readily present in the household and have been applied to treat various illnesses or protect food owing to their antimicrobial and powerful natural antioxidant properties [13]. They are well-known safe food preservatives with no adverse effects [14]. Due to the fact that universal human welfare is connected with the intake of bioactive antioxidants from natural sources, mainly from spices and medicinal herbs, spices are of great interest at present. The search for bioactive compounds with potential antioxidant properties continues to have great implications in the therapies against various free radical-mediated diseases. These compounds normally prevent oxidative reactions in cell mitochondria and protect against DNA damage and carcinogenesis. They are promising substances with an extensive series of pharmacological abilities such as anti-inflammatory, anti-bacterial, and anti-fungal properties [10,11]. The main active ingredients responsible for their antioxidant activity are polyphenols and terpenes [14]. These secondary metabolites have been proven as potential antioxidants due to their oxido-reduction properties, i.e., destroying and counteracting free radicals, and disintegrating peroxides and hydroxides [10]. Indeed, the phenolic compounds present in herbs are flavonoids. They exist in almost all herbs and are widely known to avert free radical-related injuries in various ways, preventing the formation of free radicals [11]. A limited number of spices are often used in practice for specific ailments due to their antioxidant properties. The random use of herbal drugs is not technically effective due to the absence of pharmacological toxicity testing on the host. Nonetheless, conventional spices do not initiate any host toxicity and could be candidates for the discovery and development of new antimicrobial drugs against various disease-causing organisms. Therefore, the present study was designed to assay the in vitro antioxidant and antibacterial efficiency of 15 common spices against UTI-causing pathogens.

## 2. Materials and Methods

### 2.1. Spice Extracts

About 10 g of pulverized spice material was suspended in 100 mL of methanol kept in an airtight screw-capped bottle for 3 days at 4 °C. The mixture was filtered using No.1 Whatman filter paper (Sigma Aldrich, St. Louis, MO, USA) and concentrated using a rotary evaporator at room temperature. Finally, the mixture was dissolved in 1 mL of dimethyl sulfoxide (DMSO, 10% *v/v*) and the spice extracts were stockpiled in airtight glass containers at 4 °C for further use.

### 2.2. Phytochemical Assessment of Spices

Preliminary phytochemical screening of methanolic extracts of 15 common spices was achieved by the method of Ganesan et al. [15]. Based on the analysis, the following phytochemicals were determined: phenolic compounds, alkaloids, terpenoids, carbohydrates, flavonoids, fixed oils and fats, saponins, anthraquinones, glycosides, proteins and amino acids, resins, steroids, and tannins. 

### 2.3. Quantification of Total Phenolic Content

The quantity of total phenol was assayed using spectrophotometry based on the Folin–Ciocalteu method with minor changes [16]. About 2 mL of Folin–Ciocalteau reagent was mixed with 0.2 mL of methanol spice extract and 5 mL of 20% *w/v* sodium carbonate. The mixture was maintained at 37 °C in the dark for 30 min and the optical density was read at 765 nm using a spectrophotometer. Total phenolics were determined using gallic acid as a reference. All measurements were done in replicates. Total phenolics are expressed as mg of gallic acid equiv/g of dry mass.

### 2.4. Quantification of Total Flavonoid Content

The quantity of flavonoids was measured by spectrophotometry (Spectronics India, Punjab, India) based on aluminium chloride (AlCl_3_) method following Stojanović et al. [17]. In brief, about 0.5 mL of spice extract was added to 0.5 mL of reaction medium methanol: water: acetic acid in the ratio of 14:5:1. This solutionwas added to 4 mL of AlCl_3_ reagent and kept for 5 min at 37 °C. The absorbance was read using a spectrophotometer at 430 nm. The quantity of total flavonoid is expressed as mg rutinequiv/g dry mass.

### 2.5. Antioxidant Assay

The antioxidant assay of methanolic extracts of spices was examined by following free radical scavenging techniques, namely DPPH^●^(2,2-diphenyl-1-picrylhydrazyl) assay and ABTS^●+^(2,2′-azinobis-(3-ethylbenzothiazoline-6-sulfonic acid) assay [18,19]. In addition, two reducing power methods, viz., total reducing power assay (TRP) and the iron (III) reducing antioxidant power (FRAP) assay were adopted [18,19]. 

*DPPH*^●^*assay*: Briefly, 1 mL of DPPH was added to 10 μL of the spice extract and made up to 4 mL using methanol. The contents were mixed thoroughly and incubated at 37 °C for 1 h. The optical density was read by a spectrophotometer at 517 nm.

*ABTS^●+^radical assay*: Briefly, 1 mL of ABTS solution, 1 mL of spice extract and 1 mL of potassium persulfate were taken in a test tube and the mixture was kept under dark for 24 h incubation to generate the ABTS^●+^ radical cation. The optical density was read by a spectrophotometer at 734 nm.

*TRP assay*: In short, 10 μL of the spice extract, 1 mL of potassium ferrocyanide and 1 mL of phosphate buffer were taken in a test tube and the mixture were kept in a water bath at 50 °C for 30 min. Then 1 mL of trichloroacetic acid was added to the mixture followed by centrifugation for 10 min at 3000 rpm. To 1 mL of the supernatant, 1 mL of double distilled water and 0.5 mL of FeCl_3_ were added. The mixture was mixed thoroughly and the optical density was read by a spectrophotometer at 700 nm.

*FRAP assay*: Briefly, 1 mL of spice extract was added to 1 mL of FRAP reagent, and made up to a final volume of 4 mL using distilled water. The mixture was kept in a water bath at 37 °C for 30 min. The absorbance was read at 595 nm using a spectrophotometer.

### 2.6. Collection of Urinary Pathogens

The urinary pathogens cultured in cysteine lactose electrolyte deficient agar plates were obtained from the Department of Microbiology Laboratory, Annai Teresa Biomedical Training and Research Centre, Tiruchirappalli district, India from September 2017 to December 2017. Institutional approval was obtained to collect the pathogen (ATBTRC/016, Dated: 14th June 2017) and the study was carried out in accordance with good laboratory practice (GLP). Ethical clearance was not needed since there was no animal involved in this investigation. The obtained Gram-positive and Gram-negative pathogens were streaked in MacConkey agar and blood agar (HiMedia, Mumbai, India) respectively. The plates were maintained at 37 °C for 24 h. After incubation, the organisms were kept in nutrient agar for biochemical analysis. All the bacterial pathogens were retained by weekly subcultures.

### 2.7. Biochemical Test for Confirmation of Isolated Pathogens

The bacterial pathogens were recognized and established by regular biochemical methods. Several identification experiments comprising Gram staining, motility test, methyl red test, Voges-Proskauer test, indole production test, catalase test, coagulase test, oxidase test, citrate utilization test, urease test, and triple sugar iron test were performed for the verification of the bacterial pathogens following the methods adopted by Dubey [20].

### 2.8. Isolation and Identification of UTI Pathogens

Totally 11 confirmed pathogens consisting 2 Gram-positive (*E. faecalis*, and *S. aureus*) and 9 Gram-negative (*C. freundii*, *P. vulgaris*, *E. coli*, *A. baumannii*, *P. mirabilis*, *Klebsiella oxytoca*, *K. pneumonia*, *E. aerogenes*, and *P. aeruginosa*) bacteria were used in the analysis.

### 2.9. Assay of Marketable Antibiotics Against UTI Pathogens

The antibiotic sensitivity assay was achieved using the Kirby Bauer disc diffusion method. The agar plates (Mueller–Hinton agar, Hi-Media, Mumbai, India) were used and the organisms were wiped on using sterilized cotton swab. Antibiotic discs of the most commonly recommended antibiotics such as gentamicin (10 µg), ciprofloxacin (5 µg), and norfloxacin (10 µg) were placed on the agar plate surface and the plates were maintained for 24 h at 37 °C and finally, the inhibition zone was measured. The quality of the antibiotic discs was ensured with American Type Culture Collection strains: ATCC *E. coli*-25922; *P. aeruginosa*-27853; and *S. aureus*-25923 strains. The test was performed based on the standards of antibiotic susceptibility testing recommended by Clinical and Laboratory Standard Institute (CLSI) [13].

### 2.10. Assay of Antibacterial Activity of 15 Listed Spices

Antibacterial activities of extracts of 15 common spices were performed by disc diffusion method [13]. Individual species of organism and their resistance to certain antibiotics were employed. About 6 mm thick agar was prepared for culturing of bacteria, which was punched about 6–8 wells and the lawn was incubated for 30 min. The wells were occupied with 100 μL volumes of 30 mg/mL extracts of 15 listed spices. The plates were incubated at 37 °C for 18–24 h. The antibacterial activities of 15 listed spices were determined by measuring the diameter of inhibitory zones. The experiments were carried out in triplicates and DMSO (10%) solution was used as the control.

### 2.11. MIC and MBC Determination

The MIC (minimum inhibitory concentration) and MBC (minimum bactericidal bacterial concentration) of individual spice extracts were determined. Briefly, the spice material (10 g) was immersed with methanol (100 mL) in an airtight screw-capped bottle for 3 days at 4 °C. Then the mixture was filtered, concentrated and finally thawed in 1 mL DMSO (10% *v/v*). The spice extracts were diluted with DMSO to get the concentrations of 0, 1.562, 3.125, 6.25, 12.5, 25, 50, and 10 mg/mL. To a 96-well microtiter plate, the following were added: 80 μL of diluted spice extracts, 100 μL of MH broth (Hi-Media, Mumbai, India), 20 μL of bacterial inoculum (10^9^ CFU/mL) and 5 μL of 2,3,5-triphenyl tetrazolium chloride (TTC, 0.5%) (LobaChemie, Mumbai, India). Then, the plate was incubated at 37 °C for 18 h. The appearance of pink color, which is produced by TTC showed bacterial colonies and lacking color specified the deterrence of bacterial colonies. The initial well of the plate was kept as control with no spice extract treatment. Similarly, individual well was cultured on nutrient agar and various dilutions, which had no bacterial colonies on the nutrient agar plate, represented as MBC [21].

### 2.12. Statistical Analysis

The data are given as Mean ± SD of three independent duplicates. The outcomes were matched with one-way analysis of variance (ANOVA) followed by Tukey’s test, which was employed to assess the significant variations. The variations among means at the 5% level (*p* < 0.05) were statistically significant.

## 3. Results

**Figure 1:**
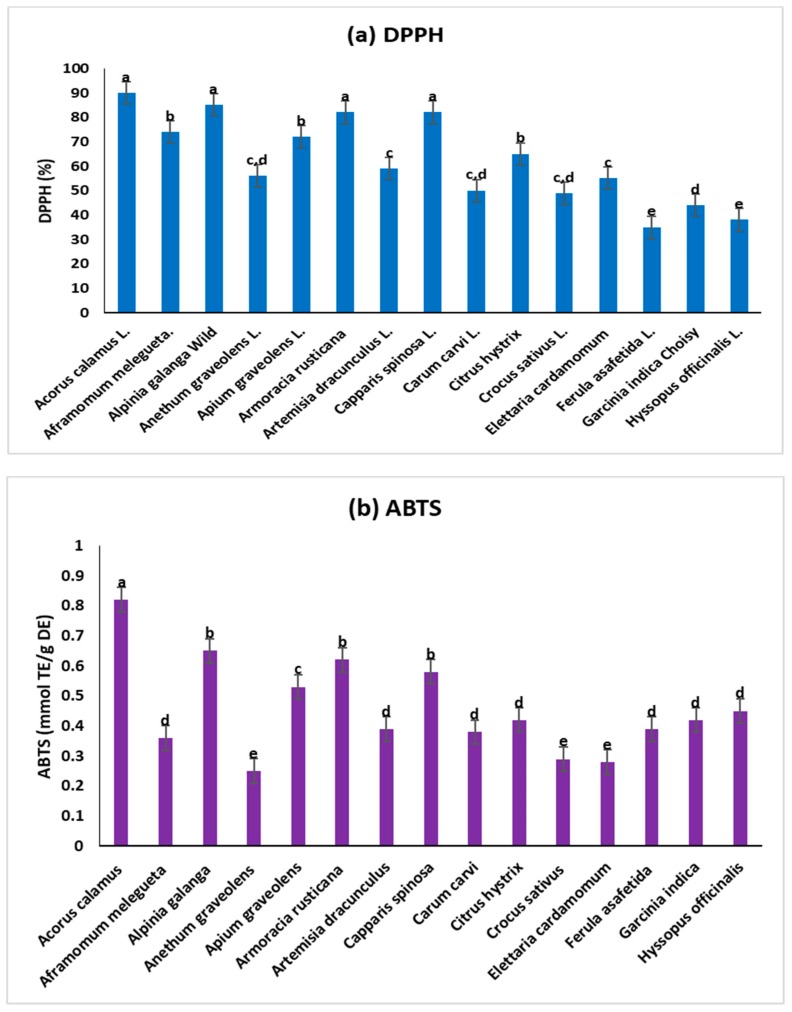

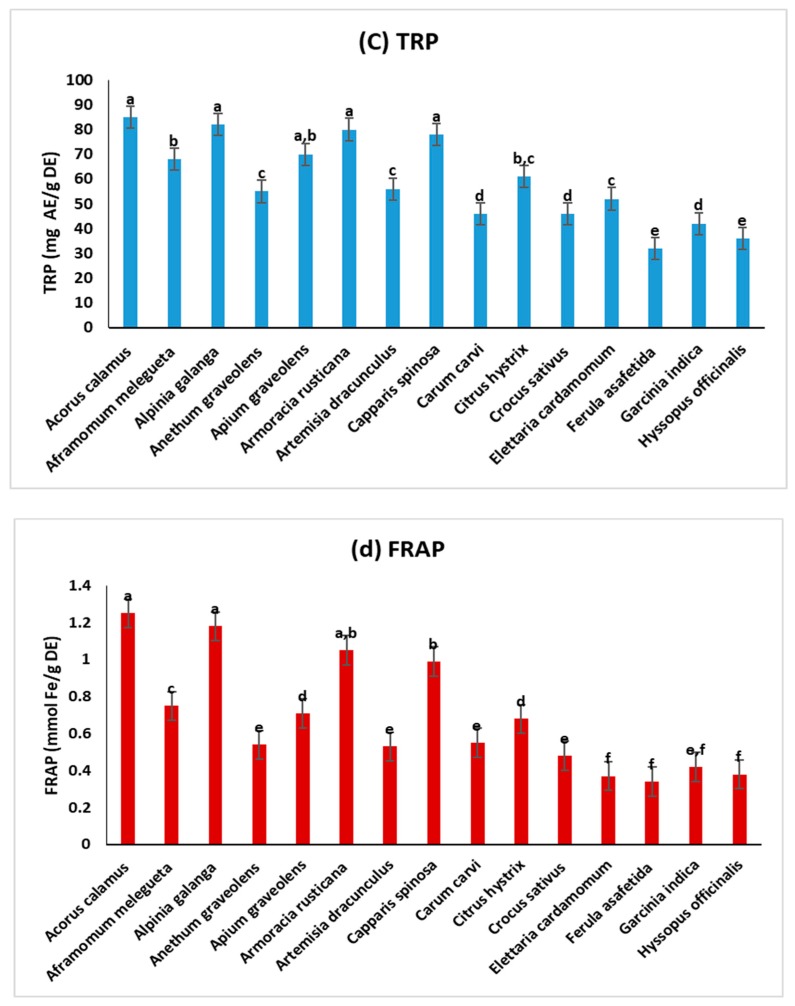
The characteristics of antioxidant potentials of methanol extracts of 15 spices. The different letters in normal bars represent the significant variations at *p* < 0.05 by using Tukey′s method. (**a**) DPPH (%), (**b**) ABTS (mmol Trolox equiv/g dry mass) (**c**) TRP (mg of ascorbic acid equiv/g dry mass), and (**d**) FRAP (mmol Fe/g dry mass).

**Figure 2:**
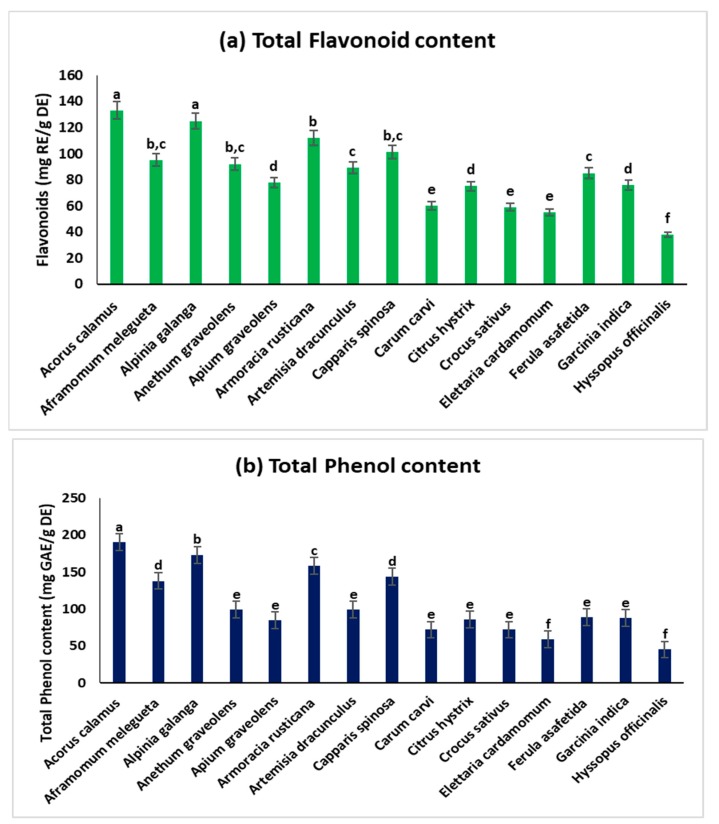
(**a**) Total flavonoid and (**b**) total polyphenol content of methanolic extracts of 15 spices. The different letters in normal bars represent the significant variations at *p* < 0.05 by using Tukey′s method The quantity of flavonoid is articulated as mg of rutin equiv/g dry mass. The quantity of polyphenol is articulated as mg of gallic acid equiv/g dry extract.

Ethnomedicinal uses of 15 common spices are shown in Table 1. All these spices are extensively used worldwide for culinary purposes as well as various infectious ailments. The qualitative phytochemical assay was performed in all those 15 spices. The spices *A. calamus*, *A. melegueta*, *A. galanga*, *A. graveolens*, *A. dracunculus*, *E. cardamomum*, *F. asafetida*, *G. indica*, and *H. officinalis* contained alkaloids, flavonoids, carbohydrates, phenolic compounds, and steroids that can be recognized to the noteworthy effect against various bacteria. The outcomes of the preliminary phytochemical assay of all listed spices are presented in Table 2. The antioxidant potential of methanolic extracts of the 15 common spices was determined by the scavenging abilities of DPPH (Figure 1a), ABTS (Figure 1b), TRB (Figure 1c), FRAP (Figure 1d), and quantities of total flavonoids (Figure 2a) and total polyphenols (Figure 2b). The outcomes of radical scavenging activities of 15 common spices from the four techniques confirmed the following order of antioxidant activity: *A. calamus > A. galangal* > *A. rusticana >* *C. spinosa* > *A. melegueta.* Similarly, the total quantities of polyphenols and flavonoids in the studied spices follow the order *A. calamus > A. galanga* > *A. rusticana > C. spinosa* > *A. melegueta.*

**Figure 3:**
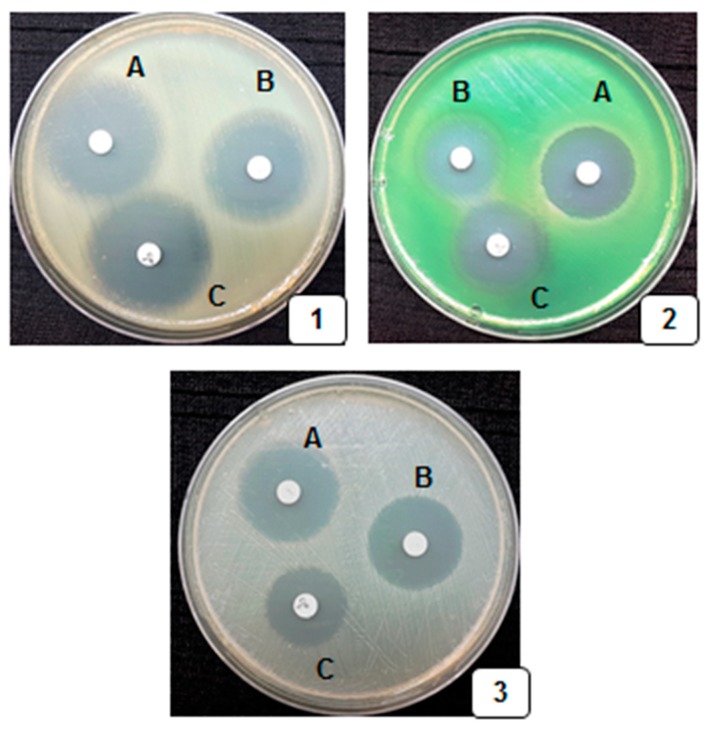
The quality control study of standard antibiotic discs by disc well diffusion method with corresponding zone of inhibitions: **A**: Norfloxacin (10 µg); **B**: Gentamicin (10 µg); and **C**: Ciprofloxacin (5 µg) with ATCC strains: (**1**) *E. coli*-25922; (**2**) *P. aeruginosa*-27853; and (**3**) *S. aureus*-25923

Various biochemical tests were employed for the validation of the test pathogens and the results are shown in Table 3. The antibacterial activity was presented using three standard antibiotics and 15 common spices against 11 UTI-causing bacteria. These tests were executed by agar-well diffusion method, and the diameters of zones of inhibition are shown in Table 4. The standard of the antibiotic discs was ensured with *P. aeruginosa*, *S. aureus* and *E. coli* strains (Figure 3 and Table 5). Extract of *A. calamus* had an inhibition zone of 27 mm against *K. oxytoca*; *A. rusticana* had a zone of inhibition of 27 mm against *P. aeruginosa*; *C. spinosa* and *A. galanga* had zones of inhibition of 27 mm and 29 mm against *P. mirabilis* respectively. The most competent eight spices possessing at least 22–27 mm as the size of the zone of circle inhibition were *A. melegueta*, *A. graveolens*, *A. rusticana*, *A. dracunculus*, *C. spinose*, *C. hystrix*, *E. cardamomum*, *G. indica* (Table 4). The four spices that attenuated a maximum number of the pathogens employed were *A. calamus*, *A. rusticana*, *C. spinosa*, and *A. galanga*, with the highest zones of inhibition of 27–29 mm. Extracts of *C. sativus* controlled *A. baumannii*. Similarly, the extracts of the seven spices *A. graveolens*, *A. graveolens*, *A. dracunculus*, *C. carvi*, *C. hystrix*, *E. cardamomum*, and *G. indica* demonstrated the control of three bacterial pathogens. The extracts of three spices, *A. melegueta*, *F. asafetida*, *H. officinalis* revealed control of three UTI-causing species. The extracts of all 15 spices and their MIC and MBC values were assessed against all listed UTI-causing bacteria and are shown in Table 6 and Table 7. *A. calamus*, *A. melegueta* ,and *C. spinosa* had the lowest MIC value of 1.51 mg/mL against *E. aerogenes*, *S. aureus*, and *P. mirabilis*. All the 15 spices had lowest MBC value (3.41 mg/mL) against a maximum number of the pathogenic bacteria. The 15 spices had the value of 21.67 mg/mL as upmost MIC and MBC against most of the bacteria. A minimal value of MIC/MBC implied that the least quantity of extracts was used, whereas an upper value indicated the use of a greater quantity of the extracts to deteriorate the bacterial colonies. According to the MIC and MBC values, the extracts of spices might be listed in diminishing order of effectiveness: *A. calamus > C. spinosa > A. melegueta > A. galangal > A. dracunculus > C. hystrix*. Among the UTI-causing bacteria, *E. coli*, followed by *A. baumannii*, *P. mirabilis, C. freundii*, *E. aerogenes*, *K. pneumoniae*, and *P. aeruginosa*, were attenuated by maximum quantities of spice extracts, as apparent from the MIC and MBC values.

## 4. Discussion

In recent years, antioxidants have attracted much attention in the field of pharmaceuticals. Drug formulations with antioxidant base are recommended for the prevention and management of many complex and metabolic ailments. Plants and their secondary metabolic derivatives are the major pioneer resources of antioxidants, which pose multiple disease healing competencies. Polyphenols are the important antioxidant resources of plants [22]. Generally, this antioxidant stuff depends on redox reactions and act as reductant, proton donor, singlet oxygen extinguisher, metal chelator, and ferryl hemoglobin reductant. These capabilities are normally related to the incidence of reductants, which deteriorate the free radicals with the donation of a proton or by prevention of peroxide formation [23,24]. As per the present evidence, the medicinal plants are rich resources of phenolic compounds including flavonoids (flavonols, flavones, isoflavones, flavonols), phenolic acids (hydroxycinnamic acid, hydroxybenzoic acid), stilbenes, coumarins, lignans, tannins, and lignins. All these compounds have innumerable biological stuff showing antioxidant activities [25,26].

In the present investigation, 15 spices have been evaluatedand found to possess antioxidant properties. Methanolic extracts of *A. calamus*, *A. galanga*, *A. rusticana*, *C. spinosa*, and *A. melegueta* demonstrated high antioxidant potential. Our study also strongly proposes antioxidant potentials of the species based on the presence of total polyphenol and total flavonoid content. Besides polyphenol, *A. calamus* has chief elements of essential oils, namely *α*-asarone, *β*-asarone, *Z*-isoelemicin, linalool, *Z*-methyl isoeugenol, shyobunone, kessane that have potent antioxidant properties [27,28]. *A. galanga* also contains essential oils such as 1’-acetoxyeugenol acetate, which has been proved as an antimicrobial as well as antioxidant agent [29]. Two flavonoids namely kaempferol and quercetin have been isolated from *A. rusticana*, that are well-known antioxidants [30]. *C. spinosa* also contains quercetin, which has been shown to possess antidiabetic, hepatoprotective and anticancer activities [31,32,33]. *A. melegueta* have essential oils and is recognized for its antidiabetic and antihypertensive effects [34]. All the tested spices contain polyphenols and flavonoids, which demonstrate the antioxidant capacity. According to recent investigations, the spices have strong antimicrobial potentials due to flavonoid and phenolic abundance [35,36,37]. These results positively correlate with the antioxidant properties and further confirms their major role in antioxidant as well as antibacterial activity of these spices. Polyphenols and flavonoids have been established to interact with the cell wall of bacteria and subsequent interference in the membrane functions or decreasing membrane fluidity [8]. Researchers have demonstrated that flavan-3-ols such as epicatechin and catechin are strongly bound to the positively charged lipid bilayer of Gram-positive bacteria; thus segregating and damaging the structure and functions of the cell, eventually causing cell death [6,36]. Another possible mechanism is inhibition of metabolic enzymes in the bacteria or interference with the synthesis of certain amino acids essential for the bacterial development [6].

In the current investigation, methanolic extracts of the four spices *A. calamus*, *A. rusticana*, *C. spinosa*, and *A. galanga* have been established to be potential in controlling a maximum number of the uropathogens. *A. calamus* has anti-inflammatory, antimicrobial, diuretic, antiurolithiatic activities and prevents several cardiovascular diseases [38]. *A. calamus* with a high content of essential oil and the key bioactive compound β-asaronehas been examined against 13 Gram-positive and negative bacteria, and three fungi [39]. *A. rusticana* contains an active principle of 5-phenyl pentyl isothiocyanate, which has been proved to show spasmolytic, antimicrobial and cytotoxic activities [40]. *C. spinosa* has been proved to exhibit anti-quorum sensing and antibiofilm potentials. This spice prevents the onset of bacterial infections and is an alternative to antibiotics [41,42]. The tuber of *A. galangal* contains pentadecane, α-humulene and 1’-acetoxyeugenol acetate, which revealed the highest antimicrobial and antioxidant activity. Therefore, it can be possibly used as a natural food preservative or as a therapeutic drug [43,44].

Urinary tract infection is the common infections positioned next to upper respiratory infection with an increasing resistance to antimicrobial drugs. These infections distress patients in all age groups and sexes [45]. Most of the UTIs are neither life-threatening nor cause any harm permanently. Multiple antimicrobial resistance among Gram-negative bacteria has been a long-term and well-known problem with UTI [46,47]. Antimicrobial resistance has been detected in several genera connected with UTI in several nations, including *E. coli*, *E. aerogenes*, *Klebsiella*, *Proteus*, *Salmonella*, *Serrata*, and *P. aeruginosa* [48,49,50,51,52]. These bacteria usually live in the gut; however, they enter into the urinary tract [45]. The occurrence of UTI is higher in women than men due to their anatomical predisposition or urothelial mucosal adherence or host factor. Furthermore, the extended spectrum of β-lactamase generating bacteria has originated in the members of Enterobacteriaceae that are the causative organisms of UTI. Other major contributing factors of UTIs are poor sanitary settings and lack of appropriate hygiene. One of the most common UTIs is leucorrhea among women, described by a whitish discharge from female genitalia [46].

The present study authenticates that all isolated uropathogens were controlled by the four most active spices namely *A. calamus*, *A. rusticana*, *C. spinosa*, and *A. galanga*, with at least 22–27 mm diameter of zone of inhibition. In general, the phytochemicals in the spices are considered very safe and have been used by conventional ethnic medicine worldwide for a long time [53]. Most of these spices are cultivated globally from many biodiversity hotspots. Apart from their aromas, savors, and tastes, these spices are identified to have numerous curative properties [54]. Recent literature has documented that the spices-based antimicrobials and related therapeutics proved their medicinal potentialities. [12,53,54]. Nowadays, the search for new antimicrobials associated with medicinal plants is taken into account drug resistance phenomenon [55,56]. These phytochemicals generally have several beneficial properties, often habitual medicine for many symptomatic illnesses [57]. For instance, *Hydrastis canadensis* does not only have antibacterial activity, but also enhances the blood circulation to the spleen [58].

The phyto-antimicrobial drugs have huge prospective demand due to the recent high antimicrobial resistance developed worldwide. In addition, the plant fractions contain the combination of varied phytochemicals [59,60,61]. Antibacterial resistance is attained in a 70S ribosome or bacterial cellular membranous obstruction [12]. The antimicrobial activity of herbs and spices involves various molecular mechanisms based on their different phytocompounds in crude form or diverse mechanisms of toxicity to the bacteria [53,55]. In the 1840s, German researchers witnessed the characteristic features of European *Oxycoccus palustris* (cranberry) and its various actions on UTIs. They found hippuric acid in the urine excreted by the individuals who consumed lots of cranberries. Generally, hippuric acid acidifies the urine and naturally prevents the UTI. Presently cranberries gained much attention in controlling or preventing UTIs [62]. Similarly, in our study, the active polyphenol or flavonoids in any form could also show bactericidal action, although pharmacokinetic/pharmacodynamic studies are still required to confirm it.

Poor personal hygiene in many females in diverse ethnic groups also increases the potential for UTI. The awareness of cleanliness among women is instantly required for certain regions of low or middle-income countries [63]. The use of spices and their phytocompounds aids in relieving many microbial infections including UTIs.

Spices are well documented as safe herbs by the American Food and Drug Administration [64], which prevent various health complaints. The current study strongly validates that all isolated uropathogens were controlled by the spices. Hence, the success of this preclinical model can be directly translated to phase I clinical trial after complete pharmacokinetic/pharmacodynamic analyses. Future clinical trials with the spices will be reliant on the path from bench to bedside that will expand patient selection and identification of biomarkers that would be critical for effective clinical progress.

## 5. Conclusions

The results of the phytochemical analysis showed that the 15 selected common spices contain potential antioxidant source of secondary metabolites such as polyphenol and flavonoids that may supply drugs for modern medicines. All 11 isolated uropathogens were controlled by the four most effective spices, namely *A. calamus*, *A. rusticana*, *C. spinosa*, and *A. galangal*. Therefore, these four spices have been proved as potent antioxidants and antimicrobial agents against UTI-causing pathogens. In addition, extraction, cleansing, and categorization of the active principles are required to ensure that the spices become novel therapeutic drugs.

## Figures and Tables

**Table 1:** Selected common spices for antibacterial activity.

S. No	Spice Name	Family	Common Name	Portions Used	Ethnomedical Practices
*1.*	*Acorus calamus* L.	Araceae	Sweet flag	Rhizome	To cure urinary infections
*2.*	*Aframomum melegueta* K. Schum.	Zingiberaceae	Grains of Paradise	Seed	To treat gastric and stomach diseases
*3.*	*Alpinia galanga* Wild	Zingiberaceae	Galanga	Rhizome	To treat asthma and respiratory infections
*4.*	*Anethum graveolens* L.	Apiaceae	Dill seed	Fruits	To treat stomach pain and throat infections
*5*	*Apium graveolens* L.	Apiaceae	Celery	Seed	To treat indigestion, stomach pain, and diarrhea
*6.*	*Armoracia rusticana* Gaerth	Brassicaceae	Horse Radish	Root	Used as an oral contraceptive and to treat bedsores, burns, and fever
*7.*	*Artemisia dracunculus* L.	Asteraceae	Tarragon	Leaf	To treat diabetes mellitus
*8.*	*Capparis spinosa* L.	Capparidaceae	Caper	Flower buds	Used to control pimples and boils during summer
*9.*	*Carum carvi* L.	Apiaceae	Caraway	Fruits	Against dysentery, bronchitis, and cough
*10.*	*Citrus hystrix* DC	Rutaceae	Kaffir Lime Leaves	Leaves	To treat stomach discomfort, diarrhea, and dyspepsia
*11.*	*Crocus sativus* L.	Iridaceae	Saffron	Parts of pistil	To control acidity and stomach disorders
*12.*	*Elettaria cardamomum* (L.) Maton	Zingiberaceae	Cardamom	Fruits	To treat bronchitis, cough, and dysentery
*13.*	*Ferula asafetida* L.	Apiaceae	Asafoetida	Resin from rhizome	To treat indigestion and toothache
*14.*	*Garcinia indica* Choisy	Clusiaceae	Kokam	Rind	To treat high acidity, sickness
*15.*	*Hyssopus officinalis* L.	Lamiaceae	Hyssop	Leaf	For stomach problems

**Table 2:** Preliminary phytochemical analysis of methanolic extracts of listed spices.

Spices	Alkaloids	Anthraquinones	Carbohydrates	Fixed Oils and Fats	Flavonoids	Glycosides	Phenolic Compounds	Proteins & Amino Acids	Resins	Saponins	Steroids	Tannins	Terpenoids
*Acorus calamus* L.	+	+	+	+	+	+	+	+	+	+	+	+	+
*Aframomum melegueta* K. Schum.	+	+	+	−	+	−	+	−	+	−	+	−	+
*Alpinia galanga* Wild	+	+	+	−	+	+	+	+	−	−	+	+	+
*Anethum graveolens* L.	+	+	+	−	+	+	+	+	+	−	+	+	−
*Apium graveolens* L.	+	+	+	+	+	+	+	+	+	+	+	+	−
*Armoracia rusticana* Gaerth	−	−	+	−	−	+	+	+	+	−	+	−	+
*Artemisia dracunculus* L.	+	+	+	+	+	+	+	−	+	−	+	+	−
*Capparis spinosa* L.	+	−	+	−	−	+	−	−	+		+	−	−
*Carum carvi* L.	−	−	+	−	−	+	+	+	+	+	+	+	+
*Citrus hystrix* DC	+	−	−	+	+	+	+	+	−	+	−	+	+
*Crocus sativus* L.	+	−	+	+	+	+	−	−	+		+	+	−
*Elettaria cardamomum* (L.) Maton	−	+	+	+	+	−	+	+	+	−	+	+	+
*Ferula asafetida* L.	+	+	+	+	+	−	+	+	+	+	+	−	−
*Garcinia indica* Choisy	+	+	+	+	+	−	+	+	+	+	+	−	−
*Hyssopus officinalis* L.	+	+	+	+	+	−	+	+	+	−	+	−	−

**Table 3:** Biochemical characters of the isolated urinary pathogens.

Tests	Grams Stain	Motility	Indole Production	Voges-Proskauer	Methyl Red	Bile Esculin	Coagulase	Citrate	Urease	Catalase	TSI	Oxidase
*Enterococcus faecalis*	+	NM	NA	NA	NA	+	NA	NA	NA	−	NA	NA
*Staphylococcus aureus*	+	NM	−	+	+	−	+	−	−	+	NA	NA
*Acinetobacter baumannii*	−	NM	−	−	−	NA	NA	+	−	+	K/K G- H_2_S-	−
*Citrobacter freundii*	−	M	−	−	+	NA	NA	+	−	+	K/A G- H_2_S+	−
*Enterobacter aerogenes*	−	M	−	+	−	NA	NA	+	−	+	K/A G- H_2_S-	−
*Escherichia coli*	−	M	+	−	+	NA	NA	−	−	+	A/A G+ H_2_S-	−
*Klebsiella oxytoca*	−	NM	−	+	−	NA	NA	+	W+	+	A/A G+ H_2_S-	−
*Klebsiella pneumoniae*	−	NM	−	+	−	NA	NA	+	+	+	A/A G+ H_2_S-	−
*Proteus mirabilis*	−	M	−	−	+	NA	NA	+	+	+	K/A G+ H_2_S+	−
*Proteus vulgaris*	−	M	+	−	+	NA	NA	−	+	+	K/A G+ H_2_S+	−
*Pseudomonas aeruginosa*	−	M	−	−	−	NA	NA	+	−	+	K/K G- H_2_S-	+

NA: Not Applicable; +: Positive; −: Negative; M: Motile; NM: Non-motile; W+: Weak positive; K: Alkaline; A: Acid; G: Gas production; H_2_S: Hydrogen sulfide.

**Table 4:** Antibacterial activities of methanolic extracts of listed spices and standard antibiotics by the disc diffusion method.

Spices	Bacteria and Their Zone of Inhibition by the List of Spices (mm)										
	*Enterococcus faecalis*	*Staphylococcus aureus*	*Acinetobacter baumannii*	*Citrobacter freundii*	*Enterobacter aerogenes*	*Escherichia coli*	*Klebsiella oxytoca*	*Klebsiella pneumoniae*	*Proteus mirabilis*	*Proteus vulgaris*	*Pseudomonas aeruginosa*
*Acorus calamus* L.	20	18	17	22	24	25	27	17	19	20	19
*Aframomum melegueta* K. Schum.	—	18	22	—	17	17	—	21	17	—	18
*Alpinia galanga* Wild	28	26	21	22	17	20	19	17	29	28	26
*Anethum graveolens* L.	—	24	16	19	24	—	22	22	26	—	24
*Apium graveolens* L.	19	22	—	—	22	—	23	15	21	19	22
*Armoracia rusticana* Gaerth	23	27	16	17	19	17	19	17	26	23	27
*Artemisia dracunculus* L.	26	23	—	21	—	26	25	—	27	26	23
*Capparis spinosa* L.	22	22	21	17	26	21	19	16	27	17	19
*Carum carvi* L.	19	17	20	19	—	17	21	—	—	19	17
*Citrus hystrix* DC	25	25	—	21	26	22	26	—	26	—	25
*Crocus sativus* L.	23	21	—	22	23	17	23	19	23	23	21
*Elettaria cardamomum* (L.) Maton	24	—	21	26	22	—	23	19	—	24	19
*Ferula asafetida* L.	—	21	12	14	—	12	—	16	21	—	21
*Garcinia indica* Choisy	22	19	—	19	—	17	15	12	26	22	—
*Hyssopus officinalis* L.	15	—	19	—	19	18	—	21	22	—	—
Gentamycin (10 µg)	20	18	17	22	24	25	19	17	19	20	19
Norfloxacin (10 µg)	19	18	22	19	17	17	25	21	17	18	18
Ciprofloxacin (5 µg)	28	26	21	22	23	20	26	17	29	28	26

**Table 5:** Quality control study of standard antibiotic discs by disc well diffusion method with reference to CLSI standard.

ID	ATCC Strains	Zone of Inhibition (mm)	CLSI Reference Range (mm)	Quality of Discs
1	*E. coli* 25922	A: 34	28–35	In range
		B: 26	19–26	In range
		C: 38	30–40	In range
2	*P. aeruginosa* 27853	A: 29	22–29	In range
		B: 23	17–23	In range
		C: 28	25–33	In range
3	*S. aureus* 25923	A: 28	17–28	In range
		B: 26	19–27	In range
		C: 24	22–30	In range

**Table 6:** MIC value of methanolic extracts of listed spices.

Spices	MIC Value (mg/mL)										
	*E. aerogenes*	*S. aureus*	*A. baumannii*	*C. freundii*	*E. faecalis*	*E. coli*	*K. oxytoca*	*K. pneumoniae*	*P. mirabilis*	*P. vulgaris*	*P. aeruginosa*
*Acorus calamus* L.	1.51	1.51	3.41	3.41	4.27	9.63	4.27	21.67	9.63	21.67	—
*Aframomum melegueta* K. Schum.	1.51	21.67	21.67	9.63	4.27	—	21.67	4.27	—	4.27	4.27
*Alpinia galanga* Wild	3.41	3.41	4.27	3.41	4.27	4.27	4.27	21.67	—	3.41	—
*Anethum graveolens* L.	—	3.41	3.41	3.41	9.63	4.27	4.27	4.27	4.27	4.27	21.67
*Apium graveolens* L.	9.63	4.27	4.27	3.41	—	—	—	21.67	4.27	—	3.41
*Armoracia rusticana* Gaerth	4.27	3.41	—	—	21.67	—	21.67	21.67	9.63	3.41	4.27
*Artemisia dracunculus* L.	3.41	3.41	3.41	3.41	4.27	4.27	3.41	21.67	3.41	3.41	21.67
*Capparis spinosa* L.	1.51	3.41	3.41	—	4.27	—	4.27	21.67	1.51	—	3.41
*Carum carvi* L.	21.67	—	—	—	4.27	—	21.67	21.67	—	21.67	4.27
*Citrus hystrix* DC	3.41	3.41	4.27	3.41	4.27	4.27	4.27	21.67	—	3.41	—
*Crocus sativus* L.	21.67	21.67	9.63	4.27	—	21.67	4.27	—	4.27	4.27	—
*Elettaria cardamomum* (L.) Maton	9.63	—	—	21.67	—	—	21.67	9.63	21.67	4.27	—
*Ferula asafetida* L.	4.27	9.63	—	—	21.67	—	—	21.67	9.63	21.67	4.27
*Garcinia indica* Choisy	—	21.67	21.67	9.63	4.27	—	21.67	4.27	—	4.27	4.27
*Hyssopus officinalis* L.	3.41	3.41	4.27	3.41	4.27	4.27	4.27	21.67	—	3.41	—

—, no activity.

**Table 7:** MBC value of methanolic extracts of listed spices.

Spices	MBC Value (mg/mL)										
	*E. aerogenes*	*S. aureus*	*A. baumannii*	*C. freundii*	*E. faecalis*	*E. coli*	*K. oxytoca*	*K. pneumoniae*	*P. mirabilis*	*P. vulgaris*	*P. aeruginosa*
*Acorus calamus* L.	3.41	9.63	—	—	21.67	—	—	21.67	9.63	21.67	4.27
*Aframomum melegueta* K. Schum.	—	21.67	21.67	9.63	4.27	3.41	21.67	4.27	—	4.27	4.27
*Alpinia galanga* Wild	3.41	3.41	4.27	3.41	4.27	4.27	4.27	21.67	—	3.41	—
*Anethum graveolens* L.	—	3.41	3.41	3.41	9.63	4.27	4.27	4.27	4.27	4.27	21.67
*Apium graveolens* L.	9.63	4.27	4.27	3.41	—	—	—	21.67	4.27	—	3.41
*Armoracia rusticana* Gaerth	4.27	3.41	—	—	21.67	—	21.67	21.67	9.63	3.41	4.27
*Artemisia dracunculus* L.	3.41	3.41	3.41	3.41	4.27	4.27	3.41	21.67	3.41	3.41	21.67
*Capparis spinosa* L.	—	3.41	3.41	—	4.27	—	4.27	21.67	—	—	3.41
*Carum carvi* L.	21.67	—	3.41	—	4.27	—	21.67	21.67	—	21.67	4.27
*Citrus hystrix* DC	3.41	3.41	4.27	3.41	4.27	4.27	4.27	21.67	—	3.41	—
*Crocus sativus* L.	21.67	21.67	9.63	4.27	3.41	21.67	4.27	—	4.27	4.27	—
*Elettaria cardamomum* (L.) Maton	9.63	3.41	—	21.67	—	—	21.67	9.63	21.67	4.27	—
*Ferula asafetida* L.	4.27	9.63	—	—	21.67	—	3.41	21.67	9.63	21.67	4.27
*Garcinia indica* Choisy	—	21.67	21.67	9.63	4.27	—	21.67	4.27	3.41	4.27	4.27
*Hyssopus officinalis* L.	3.41	3.41	4.27	3.41	4.27	4.27	4.27	21.67	—	3.41	—

—, no activity.
